# The association of breast mitogens with mammographic densities

**DOI:** 10.1038/sj.bjc.6600537

**Published:** 2002-10-07

**Authors:** N F Boyd, J Stone, L J Martin, R Jong, E Fishell, M Yaffe, G Hammond, S Minkin

**Affiliations:** Division of Epidemiology and Statistics, Ontario Cancer Institute, 610 University Avenue, Toronto, Ontario, Canada M5G 1K9; Imaging Research and the Department of Radiology, Sunnybrook and Women's College Health Sciences Centre, 2075 Bayview Ave, Toronto, Ontario, Canada M4N 3M5; Department of Radiology, Mount Sinai Hospital, 600 University Avenue, Toronto, Ontario, Canada M5G 1X5; London Regional Cancer Centre, 760 Commissioners Road East, London, Ontario, Canada N6A 5L6

**Keywords:** mammographic density, hormones, growth factors

## Abstract

Radiologically dense breast tissue (mammographic density) is strongly associated with risk of breast cancer, but the biological basis for this association is unknown. In this study we have examined the association of circulating levels of hormones and growth factors with mammographic density. A total of 382 subjects, 193 premenopausal and 189 postmenopausal, without previous breast cancer or current hormone use, were selected in each of five categories of breast density from mammography units. Risk factor information, anthropometric measures, and blood samples were obtained, and oestradiol, progesterone, sex hormone binding globulin, growth hormone, insulin-like growth factor-I and its principal binding protein, and prolactin measured. Mammograms were digitised and measured using a computer-assisted method. After adjustment for other risk factors, we found in premenopausal women that serum insulin-like growth factor-I levels, and in postmenopausal women, serum levels of prolactin, were both significantly and positively associated with per cent density. Total oestradiol and progesterone levels were unrelated to per cent density in both groups. In postmenopausal women, free oestradiol (negatively), and sex hormone binding globulin (positively), were significantly related to per cent density. These data show an association between blood levels of breast mitogens and mammographic density, and suggest a biological basis for the associated risk of breast cancer.

*British Journal of Cancer* (2002) **87**, 876–882. doi:10.1038/sj.bjc.6600537 www.bjcancer.com

© 2002 Cancer Research UK

## 

Differences between individuals of the same age in the radiological appearance of the breast reflect differences in tissue composition. Epithelial and stromal tissues attenuate X-rays more than does fat, and appear light on a mammogram, while fat appears dark (Johns and Yaffe, 1987). Extensive areas of light appearing tissue on a mammogram, referred to as ‘mammographic density’, have been found to be associated with 4–6-fold differences in the risk of breast cancer, after taking into account other known risk factors for the disease (Boyd *et al*, 1998).

The biological basis for inter-individual variations in breast tissue composition, and for the association of these variations with risk of breast cancer is, however, unknown, although several factors are known to influence the radiographic appearance of the breast. Mammographic density decreases with greater parity, greater body weight, greater age (Boyd *et al*, 1998), and is increased by hormone replacement therapy (Lundström *et al*, 1999), and decreased by Tamoxifen (Atkinson *et al*, 1999; Brisson *et al*, 2000). All of these observations suggest that variations in exposure to endogenous hormones or growth factors may be responsible for the differences in breast tissue composition that are reflected in differences in the extent of mammographic density.

The purpose of the present study was to examine, in a cross-sectional study design, the association between mammographic density and blood levels of endogenous hormones and growth factors. The hormones examined include total and free oestradiol and progesterone, sex hormone binding globulin (SHBG), growth hormone, insulin like growth factor-I (IGF-I), its principal binding protein - insulin-like growth factor binding protein-3 (IGFBP-3), and prolactin.

## METHODS

### General method

The general method employed was to select pre- and postmenopausal women without breast cancer but with different degrees of mammographic density, to collect information from them about risk factors, and to measure hormones and growth factors in blood samples obtained under standardised conditions. We determined the total area of the breast, and the area of dense tissue on mammography, using a previously described computer assisted method (Byng *et al*, 1994).

### Method of sampling and classification of breast density

#### Source of subjects

The goal of the sampling procedure was to assemble premenopausal and postmenopausal women with a wide spectrum of mammographic density. Between 1994 and 1997 subjects were identified from the mammographic units of St. Michael's, Women's College and Mount Sinai Hospitals in Toronto. Women were referred to these units for mammographic examination for a variety of reasons, including suspicion of breast disease, the presence of risk factors such as a family history of breast cancer, or for routine examination. Because per cent mammographic density is a continuous variable, we wished to analyse it as such, without division into arbitrary categories. However, it was impractical to measure all mammograms in the participating institutions. We therefore used in each hospital the extent of mammographic density, expressed as a percentage of the breast area (<10%, 10<25%, 25<50%, 50<75%, and ⩾75%), that was recorded by radiologists during film reporting. We wished to ensure that we recruited women with as wide a range of mammographic density as possible. The number of subjects in each of the five radiological categories of density that were actually recruited were as follows: <10% *n*=101, 10<25% *n*=62, 25<50%, *n*=60, 50<75%, *n*=60, and ⩾75%, *n*=99.

Breast density was subsequently definitively classified as a continuous variable by quantitative methods that are described below. On hundred and fifty-five subjects (41%) fell in the same category according to the two methods of classification, and 317 (83%) were within one category on both methods. (The means of the continuous measure according to quintiles are shown in Table 3A,B

**Table 3:**
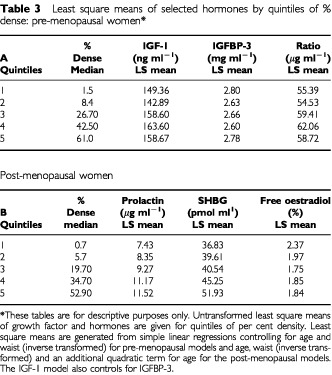
Least square means of selected hormones by quintiles of % dense: pre-menopausal women*

.)

#### Method of recruitment

Subjects identified in the manner described above were sent a letter, with an explanation of the goals and procedures of the study, followed by a phone call, during which their eligibility was determined. Premenopausal women were eligible for the study if they were menstruating regularly, were not pregnant or breastfeeding, and had not had a hysterectomy or oophorectomy. Postmenopausal subjects were eligible if they had spontaneous amenorrhoea for at least 12 months, or had a hysterectomy and were aged 50 or more, or had a bilateral oopherectomay at any age. All subjects taking any type of exogenous hormone preparation in the previous 6 months, or who had breast augmentation or reduction, or a previous history of breast cancer, or were under investigation for breast cancer, were excluded.

Eighteen hundred and seventy-four recruitment letters were mailed, and we were subsequently able to contact 1134 by telephone. Of these, 526 were determined to be not eligible, and 174 stated they were not interested before their eligibility could be determined. Of the remaining 434 who were eligible, 382 agreed to participate in the study. Thus, 88% of subjects who were contacted, and found to be eligible (approximately 34% of all subjects who could be contacted), agreed to take part in the study, and the rate of participation did not vary according to the extent of mammographic density.

### Measurements

Subjects who agreed to enter the study were then visited in their homes by the study research assistant. These visits took place in the morning after subjects had fasted for at least 12 h. All measurements in premenopausal women were made when subjects were in the luteal phase of the menstrual cycle (between days 20–24). Blood was refrigerated immediately after collection, spun and serum separated within 1–2 h of collection, and stored at −70°C until analysis. The mammogram closest to the time of the blood draw was used, and the average time interval between the mammogram and the blood draw was 32 weeks. The phase of the menstrual cycle at which mammograms were obtained is, however, not known because subjects were recruited into the present study after the mammograms had been taken.

#### Epidemiological data

Information about epidemiologic risk factors for breast cancer, and other factors, was collected using a questionnaire, developed for this purpose, that included questions about menstrual and reproductive history, and family history of breast and other cancer.

#### Anthropometric measures

Each subject was weighed on a balance scale and measured for height. Skinfold thickness in the triceps, sub-scapular, and iliac crest areas was measured using Lange calipers, and waist and hip circumferences were measured with a tape measure. The research assistant who made these measurements was trained and certified by the Department of Athletics and Recreation, University of Toronto.

### Measurement of blood samples

Serum oestradiol (in premenopausal women) and progesterone levels were measured using radioimmunassays kits purchased from Orion Diagnostica (Oulunsalo, Finland), and the inter-assay performance was monitored using controls provided by the kit manufacturer. Oestradiol in serum from postmenopausal women was measured by Esoterix Laboratories, California, USA by radioimmunoassay after extraction.

Serum SHBG levels were determined using an established steroid-binding capacity assay using reference serum samples to control for inter-assay performance (Hammond and Lähteenmäki, 1983). The percentages of free oestradiol in serum samples were estimated from a nomogram describing the relationship between serum SHBG levels and percentages of free oestradiol measured using the centrifugal ultrafiltration-dialysis method in a reference population of normal weight pre- and postmenopausal women (Langley *et al*, 1985). These values were then used to calculate the free oestradiol concentrations in serum samples from the total oestradiol measurements.

IGF-I, IGFBP-3 and growth hormone were all measured by Esoterix, California, USA, IGF-I and IGFBP-3 using a competitive binding radioimmunoassay, and growth hormone with a two site immunometric assay. Prolactin was measured by radioimmunoassay in the Wellesley Hospital in Toronto. The per cent coefficient of variation within assays was less than 7% for all (except progesterone which was 8.7%), and between assays was less than 10% for all (except progesterone which was 11.9%).

#### Definitive classification of breast parenchymal pattern

The measurements that are the subject of the analyses that follow were made using a randomly selected, craniocaudal mammographic view of one breast from each subject.

Mammograms were digitised using a Lumisys model 85, which provides up to 12 bit density resolution and 50 μm spatial resolution, and presented for analysis as an array of 675×925 pixels (0.067 mm^2^ per pixel). Images were analysed using a computer assisted method (Byng *et al*, 1994, 1996a,b). Images from subjects in the pre and postmenopausal groups were randomly ordered, and thus measured without knowledge of menopausal status, or of any of the other characteristics of the subjects.

The observer selected a threshold grey value to separate the image of the breast from the background, and a second threshold to identify the edges of region(s) which were representative of radiographically dense tissue. We then calculated the histogram of pixel values within these boundaries, and calculated the size of the projected area of the breast in the image, and of the area of density. The percentage of radiographic density is the area of dense tissue divided by the entire projected area of the breast and multiplied by 100.

A subset of duplicate images were included as a check on reliability. Reliability of the measurements made in the present study was high with a test-retest correlation of at least 0.9.

#### Statistical analysis

Data analysis was carried out using the SAS statistical software package (Sas Institute Inc, (1989)). Data were inspected for normality before analysis and, when necessary, a transformation was applied. The distributions of many of the hormone measurements, and, as a consequence of our recruitment method, of the mammographic measures, were not normal and transformations were required. Details of the transformations used are given in the footnotes of the tables of results. Multiple regression analysis was used to examine the association between mammographic measurements and blood levels of hormones and growth factors after adjustment for other variables. The residuals from these analyses were plotted against their predicted values and inspected for patterns to ensure that the transformations were appropriate.

Values for growth hormone were undetectable in 128 of the 382 subjects (34%) and for purposes of analysis were assigned the value of 0.2 ng l^−1^, the lower limit of sensitivity for the assay. An additional 22 observations had missing growth hormone information (19 postmenopausal, 3 premenopausal). There was, however, no relationship between mammographic density and whether growth hormone could be detected.

To examine the relationships between hormones, growth factors and mammographic measures we carried out a series of multiple regression analyses that adjusted sequentially for other factors that could be confounding or modifying variables. For two variables, IGF-I and total oestrogen, their respective binding proteins, IGFBP-3 and SHBG, are known to determine the amount of free and biologically active growth factor or hormone, and were introduced into the regression analyses before the other variables. Among the other factors examined, that included other risk factors for breast cancer (age, age at menarche, parity, age at first live birth, number of live births, and a family history of breast cancer), measures of body size, and blood levels of other hormones or growth factors, only age and measures of body size had a substantial influence on the results. Among the measures of body size examined, that included height, weight, body mass index, waist and hip circumference and their ratio, and skinfold thickness measurements, the waist measurement was the most strongly correlated with mammographic density, and was the measure of body size used in these analyses. In all of these analyses both mammographic measures, and blood levels of hormones and growth factors, were treated as continuous variables.

## RESULTS

### Characteristics of subjects

Table 1

**Table 1:**
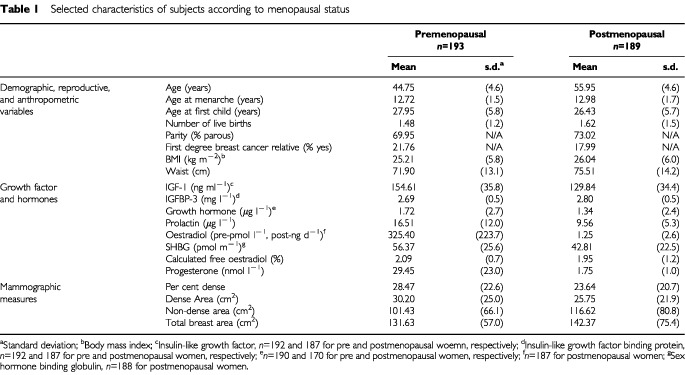
Selected characteristics of subjects according to menopausal status

describes the pre and postmenopausal subjects studied according to the distribution of selected risk factors for breast cancer and anthropometric variables. There were 193 premenopausal, and 189 postmenopausal subjects. The mean age was 45 years for premenopausal women, and 56 years for the postmenopausal. The average time since menopause in postmenopausal subjects was 7 years. About 22% of pre and 18% of postmenopausal subjects had at least one first degree relative with breast cancer. Mean blood levels of IGF-I, growth hormone and prolactin were all lower in postmenopausal than in premenopausal subjects. Mean per cent breast density and the area of dense tissue were both lower, and the areas of non-dense tissue and total area were greater in the postmenopausal.

Sixty subjects had previously taken hormone replacement therapy (HRT), stopping an average of 5 years before the date of the mammogram. After adjustment for age, the mean per cent density was similar in those who had previously used HRT and in those who had not, in both premenopausal (respectively 27.3, *n*=13, and 28.5, *n*=180) and postmenopausal subjects (respectively 24.8, *n*=47, and 23.3, *n*=142).

### Relationship between growth factors, hormones and mammographic measures

Table 2

**Table 2:**
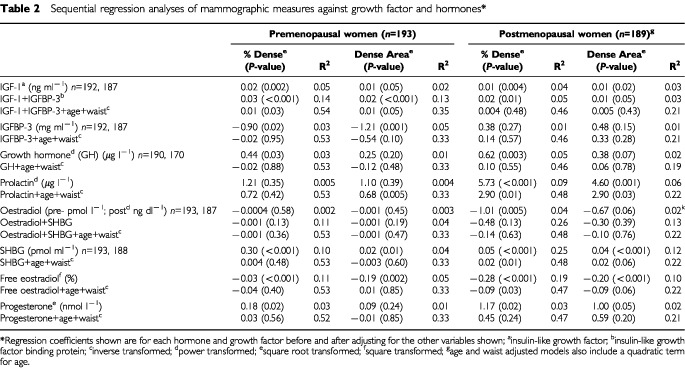
Sequential regression analyses of mammographic measures against growth factor and hormones*

shows the results of sequential regression analyses of mammographic densities against IGF-I and IGFBP-3, hormones and SHBG. There were 187 premenopausal, and 165 postmenopausal women, with complete data for all of these variables. Results are shown for both per cent mammographic density, and for the area of dense tissue in the mammogram, as this reflects the amount of stromal and epithelial tissue in the breast.

In premenopausal women, IGF-I was significantly associated with per cent mammographic density before and after adjustment for IGFBP-3, age and the waist measure. Similar associations were seen between IGF-I and the area of dense tissue, although these were of borderline statistical significance after adjustment was made for age and waist. In premenopausal women, IGF-I explained 5% of the variance in per cent density. In postmenopausal subjects, IGF-I was significantly associated with both mammographic measures before and after adjustment for IGFBP-3, but significance was lost after the introduction of age and the waist measurement into the model.

In premenopausal women only, IGFBP-3 was significantly negatively associated with per cent density and dense area before but not after adjustment for age and waist.

Growth hormone levels, in both pre and postmenopausal women, were significantly and positively associated with per cent density, where they accounted for 3–5% of the variance, but these associations were no longer significant in either group after adjustment for age and waist. Prolactin levels were not significantly associated with either of the mammographic measures in premenopausal subjects, but were positively and significantly associated with both measures in postmenopausal women, before and after adjustment for age and waist. Prolactin accounted for 9% of the variance in per cent density, and 6% of the variance in dense area, in the postmenopausal.

Total oestradiol was not associated with either mammographic measure in the premenopausal, but was negatively associated with per cent density in postmenopausal women, before, but not after adjustment for SHBG. SHBG was significantly positively associated with both mammographic measures in pre and postmenopausal subjects, but remained significant after adjustment for the waist measure only in the postmenopausal. SHBG accounted for 25% of the variance in per cent density in postmenopausal women, but only 10% in the premenopausal.

Progesterone was significantly and positively associated with per cent density in both pre and postmenopausal women, but became non-significant in both groups after adjustment for age and the waist measure. (Our intention was to obtain blood samples in premenopausal women during the luteal phase of the menstrual cycle and 83% of subjects had progesterone levels of 5 nmol l^−1^ or greater indicating that ovulation had occurred). Free oestradiol, calculated from SHBG levels, showed negative associations with per cent density that remained statistically significant after adjustment only in the postmenopausal.

The adjusted least square mean levels of the hormones and growth factors according to quintiles of per cent density are shown in Table 3, for those hormones that remained statistically significant after adjustment for all variables, as well as for IGFBP-3. This table shows untransformed values and they are shown for descriptive purposes only, to illustrate the magnitudes of the associations found. In premenopausal women, IGF-I levels were 6% higher, IGFBP-3 levels 1% lower, and their ratio 6% higher in fifth quintile (median per cent density=61%) compared to the first (median per cent density=1.5%). In postmenopausal women, prolactin levels were 36% higher in the fifth quintile (median per cent density=52.9%), relative to the first (median per cent density=0.7%). Also in the postmenopausal, SHBG levels were 29% higher in the fifth quintile, and calculated free oestradiol levels were 22% lower in the fifth quintile of per cent density compared to the first.

## DISCUSSION

The proportion of the mammogram occupied by radiologically dense tissue reflects the proportion of the breast that is comprised of epithelium and/or stroma, and is strongly related to risk of breast cancer (Boyd *et al*, 1998). These results show that four factors that have effects on cell proliferation in the breast are associated with variations in mammographic densities. Before adjustment for other risk factors, IGF-I and growth hormone, both mitogens in the breast (Yee, 1998), were both positively associated with per cent mammographic density in premenopausal women, and IGFBP-3, an inhibitor of cell proliferation that also promotes apoptosis (Yee *et al*, 1991), was negatively associated with density. In postmenopausal women, IGF-I, growth hormone and prolactin, also a mitogen (Friesen, 1976), were associated positively with per cent density. After adjustment for the waist measurement, only IGF-I in the premenopausal, and prolactin in the postmenopausal, remained significantly associated with per cent density. After adjustment, total oestradiol was not significantly associated with mammographic density in either menopausal group. Although a larger study would have greater statistical power to detect associations, those seen here between mammographic density and total oestradiol were negative in direction. In postmenopausal women, per cent density was strongly and positively associated with SHBG, and was negatively associated with free oestradiol. Blood levels of SHBG explained more variance in per cent mammographic density than any of the other hormones or growth factors measured here.

The time interval between the mammogram and blood collection was relatively short, an average of 5 months, and blood levels of progesterone confirmed that blood collection was carried out in the luteal phase of the menstrual cycle in more than 80% of premenopausal women, but the phase of the cycle at the time of the mammogram was not known. However, the coefficient of variation for per cent density was the same in premenopausal and postmenopausal subjects (respectively 79.2 and 87.6), suggesting that, after allowance has been made for the difference in mean level, the menstrual cycle was not a major source of variation in per cent density in premenopausal subjects.

These results in part confirm the findings of a cross-sectional study in the Nurses Health Study (NHS) by Byrne *et al* (2000) which showed that blood levels of IGF-I were correlated positively, and IGFBP-3 negatively, with per cent mammographic density in premenopausal but not in postmenopausal women. We have found in previous work, using immunohistochemistry in breast biopsies, that the area of tissue stained for IGF-I is associated with mammographic density in women under the age of 50, but not in older women (Guo *et al*, 2001). It has also been shown in a cohort study, carried out in the NHS, that higher blood levels of IGF-I were related to the subsequent development of breast cancer in premenopausal but not postmenopausal women (Hankinson *et al*, 1998). A further study in the NHS found higher blood levels of prolactin to be associated with an increased risk of breast cancer in postmenopausal, but not in premenopausal women (Hankinson *et al*, 1999).

The associations of IGF-I and prolactin with risk of breast cancer in the NHS, respectively in pre and postmenopausal women, are thus similar to the associations with mammographic densities according to menopausal status seen in the present study. The average age of the premenopausal women studied here was 45 years, and some may have been perimenopausal. The association of growth factors and hormones at earlier ages is at present unknown but it is known that blood levels of IGF-I peak in adolescence (Massa *et al*, 1992; Cara *et al*, 1987), and that mammographic density is also greatest in early life (Hart *et al*, 1989).

Because of the known biological activities of IGF-I, growth hormone and prolactin, and their associations with breast cancer in humans and/or animal systems, these data suggest potential biological mechanisms for the association of mammographic densities with risk of breast cancer. There is increasing evidence that the growth hormone-IGF-I axis plays a role in carcinogenesis in the breast (Pollak, 1998; Ruan *et al*, 1992) and transgenic mice that over-express IGF-I or growth hormone have an increased incidence of mammary cancer (Tornell *et al*, 1992; Macaulay, 1992; Ruan *et al*, 1992; Yee, 1998). The administration of growth hormone to ageing female monkeys induced hyperplasia of the mammary epithelium, with a striking increase in the number and cellularity of lobules, and an increase in the mitotic labelling index (Ng *et al*, 1997). Further, similar factors influence mammographic densities, and the hormones and growth factors found here to be associated with them. Breast density decreases with increasing age, after pregnancy, and at the menopause (Brisson *et al*, 1982; de Waard *et al*, 1984; Grove *et al*, 1985; Byrne *et al*, 1995). Blood levels of growth hormone, IGF-I and prolactin also decline with increasing age (Hall *et al*, 1999). Prolactin levels are also permanently reduced by pregnancy, and are lower after the menopause than before (Reyes *et al*, 1977; Wang *et al*, 1988; Metka *et al*, 1995; Nasu *et al*, 1997).

However, in contrast to the lack of association between total oestradiol and mammographic densities observed here, higher blood levels of total oestradiol have been found in postmenopausal women to be associated with an increased risk of breast cancer (Toniolo *et al*, 1995; Cauley *et al*, 1999). Further, oestrogen replacement therapy (ERT) increases breast density in some subjects, particularly when combined with a progestin (Rutter *et al*, 2001), and Tamoxifen has been shown to decrease mammographic densities (Atkinson *et al*, 1999; Brisson *et al*, 2000; Chow *et al*, 2000). However, these agents also affect the growth hormone-IGF-I axis. ERT increases blood levels of growth hormone, and reduces blood levels of IGF-I (Andersen *et al*, 1980), and Tamoxifen reduces secretion of growth hormone (Malaab *et al*, 1992), and lowers blood levels of IGF-I, at least in postmenopausal women (Pollak *et al*, 1990).

Mammographic densities in postmenopausal women were surprisingly associated positively with serum SHBG levels, and negatively with serum free oestradiol levels. These data imply that any oestrogenic effects on breast density are not related to the non-protein bound or ‘free’ fraction of oestradiol in the circulation, that is generally considered to be the biologically active form. It is possible therefore that SHBG itself may help to promote the effects of oestradiol in more direct ways, for instance by interacting with plasma membrane binding sites in target cells within the breast (Fortunati *et al*, 1999).

It is uncertain whether the associations found here between mammographic densities and blood levels of IGF-I, prolactin and SHBG are the result of one, or more than one, underlying mechanism(s). Some of the observed features may be explained by the actions of growth hormone. IGF-I is produced in the liver and other tissues, including the breast, in response to growth hormone (Herington, 1991), and blood levels of both IGF-I and prolactin are elevated in transgenic mice that over-express growth hormone (Bartke *et al*, 1999). Blood levels of SHBG could be influenced indirectly by growth hormone, through their common association with body fat stores, which are in part regulated by growth hormone (Wabitsch *et al*, 1995; Ng *et al*, 1997; Considine, 1997), and are negatively correlated with SHBG (Plymate *et al*, 1990) The strong negative associations observed in these data between the mammographic measures of per cent density and dense area, and the waist measurement, may thus represent separate biological effects of growth hormone respectively on the proliferation of breast tissue, and on the mobilisation of fat stores.

An alternative potential explanation for these findings may lie in the known actions of oestrogen. Oestrogen is involved in the control of the secretion of both growth hormone (Malaab *et al*, 1992; Veldhuis, 1996; Veldhuis *et al*, 2001) and prolactin (Yen *et al*, 1974; Adashi *et al*, 1980; Gudelsky *et al*, 1981), and regulates the synthesis and blood levels of SHBG (Loukovaara *et al*, 1995; Pasquali *et al*, 1997). Although blood levels of oestradiol were not positively associated with mammographic density in the present study, other forms of oestrogen not measured here, including oestrogen metabolites, might be responsible for the observed findings.

These findings suggest novel potential approaches to breast cancer prevention, directed at modulation of the growth hormone-IGF-I axis and prolactin. Furthermore, unexpected relationships between the determinants of serum free oestradiol levels and mammographic densities suggest that manipulation of SHBG levels or function could also be beneficial. There is clearly a need for an improved understanding of the genetic and environmental factors that influence levels of these hormones and growth factors, and in particular the relationship between these factors and other known influences on risk of breast cancer, including familial, menstrual and reproductive risk factors, and ovarian hormones.
